# Remaining challenges in the diagnosis of early stage cardiac sarcoidosis

**DOI:** 10.1002/ccr3.2114

**Published:** 2019-04-12

**Authors:** Takahiro Tsushima, Shunsuke Sasaki, Satoshi Yuda, Masayuki Ohta, Ivor Cammack, Hiroyuki Sato, Kentaro Hayashi, Mitsugu Hirokami

**Affiliations:** ^1^ Department of Medicine Case Western Reserve University, University Hospitals Cleveland Medical Center Cleveland Ohio; ^2^ Division of Cardiology Teine Keijinkai Hospital Sapporo Japan; ^3^ Department of General Internal Medicine Teine Keijinkai Hospital Sapporo Japan

**Keywords:** ^18^F‐FDG‐PET (^18^F‐Fluorodeoxyglucose positron emission tomography), cardiac magnetic resonance imaging, cardiac sarcoidosis, complete atrioventricular block, negative biopsy, speckle tracking echocardiography, transthoracic echocardiography

## Abstract

Despite the requirement for histopathological evidence to make a definite diagnosis of cardiac sarcoidosis, the sensitivity of endomyocardial biopsy is still low. Recently, Japanese Circulation Society suggests a new strategy that patients diagnosed clinically do not require the endomyocardial biopsy evidence. Physicians should familiarize themselves with such paradigm shifts.

## 1 CASE REPORT

Although many studies have reported immune‐suppression produces better outcomes for early stage cardiac sarcoidosis, it is often difficult to obtain early histopathological evidence. This case highlights the importance of clinically diagnosing and treating cardiac sarcoidosis prior to histopathological diagnosis, as suggested by the Japanese Circulation Society.

**Figure 1:**
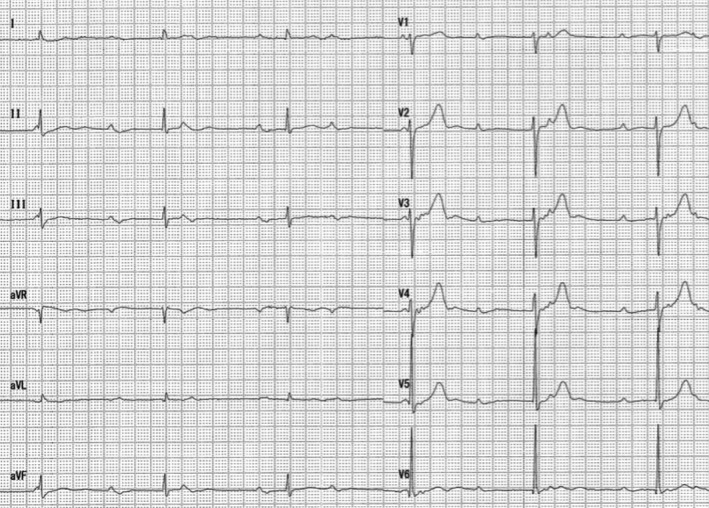
Electrocardiogram on admission

**Figure 2:**
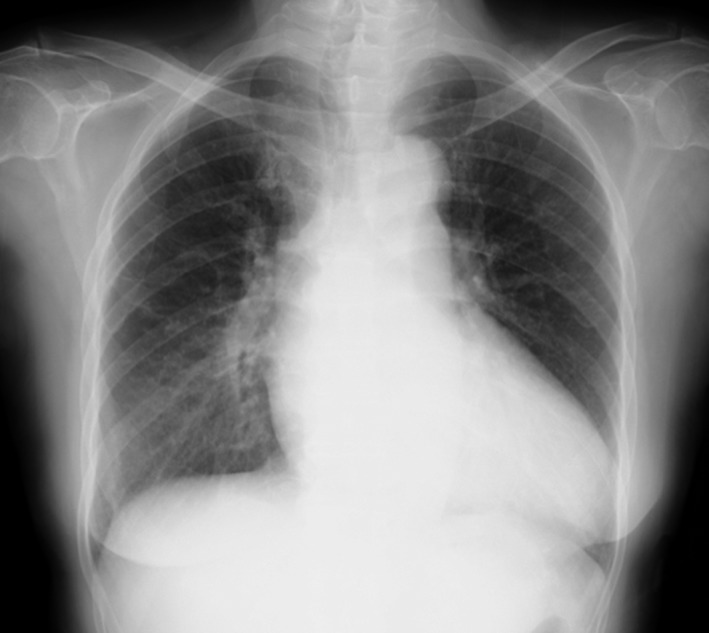
Chest X‐ray on admission

A 73‐year‐old woman with chronic hypertension presented with a one‐week history of gradually progressive exertional dyspnea and episodic lightheadedness. Her blood pressure on arrival was 147/73 mm Hg and heart rate was 39 beats per minute. Her physical examination was notable for jugular venous distention, a holosystolic heart murmur, and bilateral coarse crackles on auscultation. Electrocardiogram showed complete atrioventricular block (CAVB) without ischemic changes (Figure 1). She was not on any medications likely to cause CAVB and initial blood tests did not show any electrolyte abnormalities. Her initial cardiac biomarkers were elevated: troponin‐I was 269.7 pg/mL (reference range: <26.0) and brain natriuretic peptide was 265.2 pg/mL (<18.4). Chest X‐ray (CXR) showed stage II bilateral hilar lymphadenopathy (BHL) and pulmonary edema (Figure 2). She underwent a transvenous temporary pacing for symptomatic CAVB and was admitted to cardiology. The coexistence of BHL and acute development of CAVB without obvious reversible etiology fostered the suspicion of cardiac sarcoidosis (CS). However, transthoracic echocardiography (TTE) lacked CS‐specific findings such as regional wall motion abnormalities or basal interventricular septal wall thinness. Using speckle tracking echocardiography (STE), both longitudinal and circumferential strain values were within the normal range. There were no ophthalmological or dermatological manifestations of sarcoidosis. Lymph node biopsies from both subcutaneous lymph node and endobronchial ultrasound‐guided transbronchial needle aspiration (EBUS‐TBNA) were negative. During this time, her CAVB remained intermittent. Before performing permanent pacemaker (PPM) implantation, we planned endomyocardial biopsy (EMB) from the right ventricle along with cardiac magnetic resonance imaging (CMR). To allow these investigations, we removed the temporary pacing lead after confirming her intrinsic heart rate was adequate and stable. However, she developed frequent presyncopal episodes and PPM was implanted without obtaining EMB and CMR.

**Figure 3:**
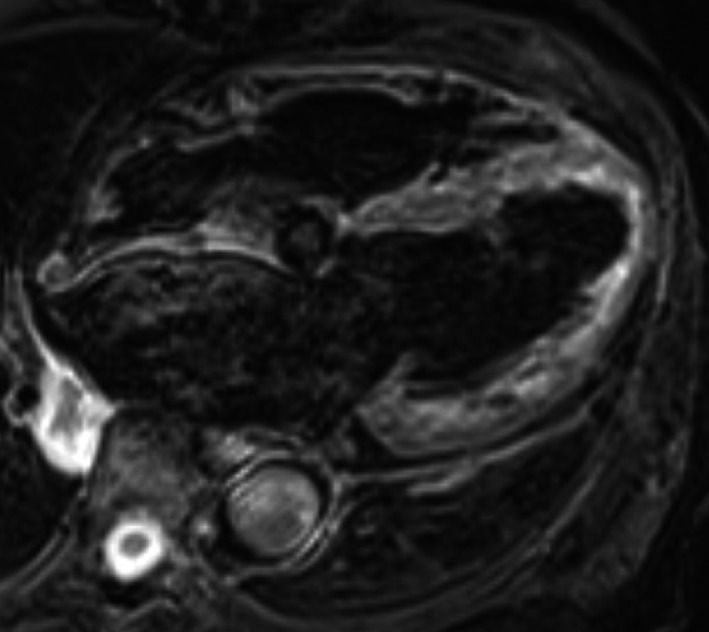
Longitudinal cardiac magnetic resonance with short tau inversion recovery (STIR)

**Figure 4:**
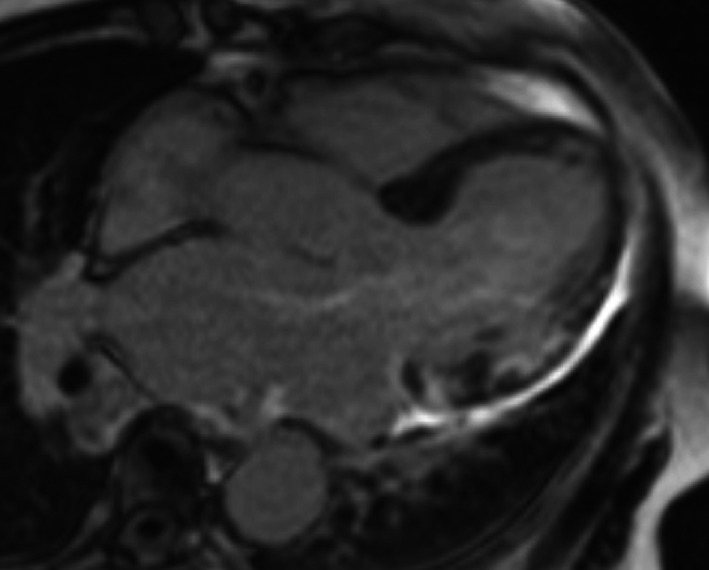
Late gadolinium enhancement (LGE) with longitudinal cardiac magnetic resonance imaging

**Figure 5:**
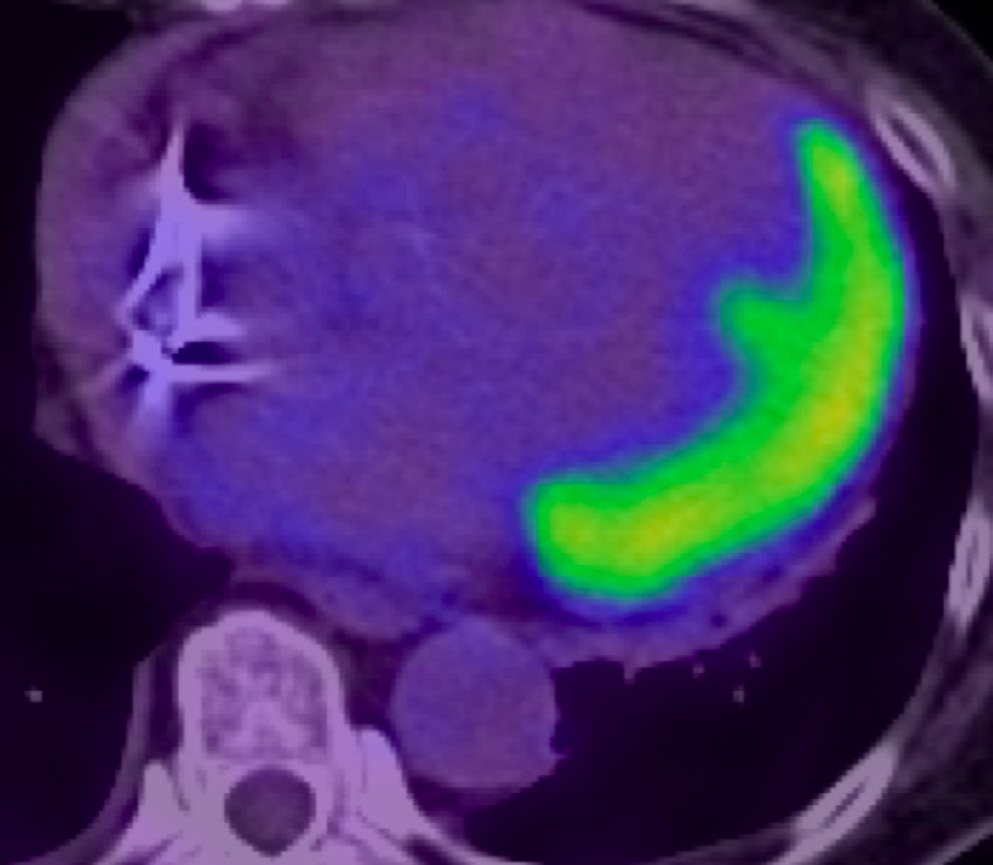
^18^F‐Fluorodeoxyglucose positron emission tomography (^18^F‐FDG‐PET) on admission (cardiac uptake value: SUV max 7.8)

**Figure 6:**
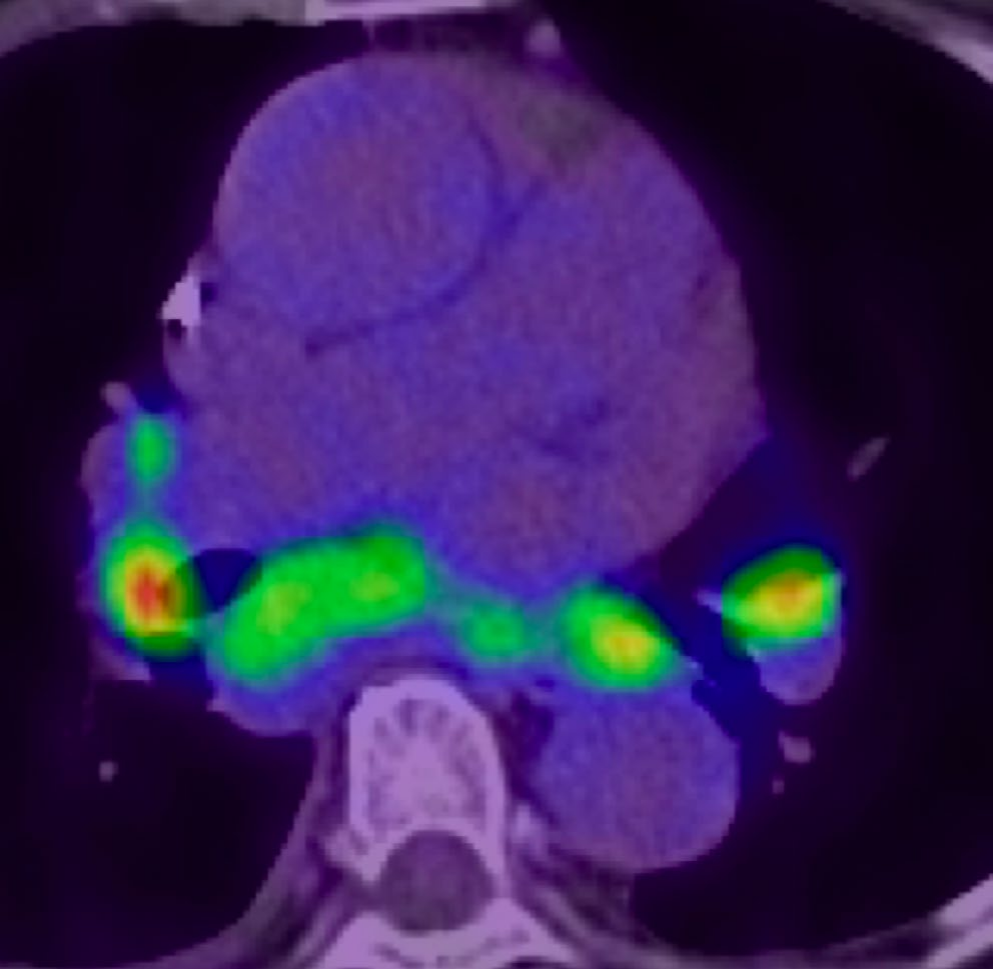
^18^F‐Fluorodeoxyglucose positron emission tomography (^18^F‐FDG‐PET) on admission (pulmonary and hilar lymph node uptake value: SUV max 12.5)

We performed CMR once the exempt period for conditionality had lapsed after PPM implantation, which demonstrated extensive myocardial edema on short tau inversion recovery and multifocal late gadolinium enhancement (LGE) in the left ventricle (LV) (Figures 3 and 4). In addition, ^18^F‐Fluorodeoxyglucose positron emission tomography (FDG‐PET) showed extensive multifocal uptake in mediastinal and hilar lymph nodes, and the left ventricle (Figures 5 and 6). Her lymphadenopathy was limited to the mediastinal space. Bloods showed a mildly raised interleukin‐2 receptor at 553 U/mL (reference range: 122‐496), but lactate dehydrogenase was within normal limits. Based on these findings, our hematology team had a low suspicion of lymphoproliferative disease or malignant lymphoma and we made a final assessment of “clinically‐diagnosed cardiac sarcoidosis” based on the Japanese Circulation Society (JCS) guideline.^1^

**Figure 7:**
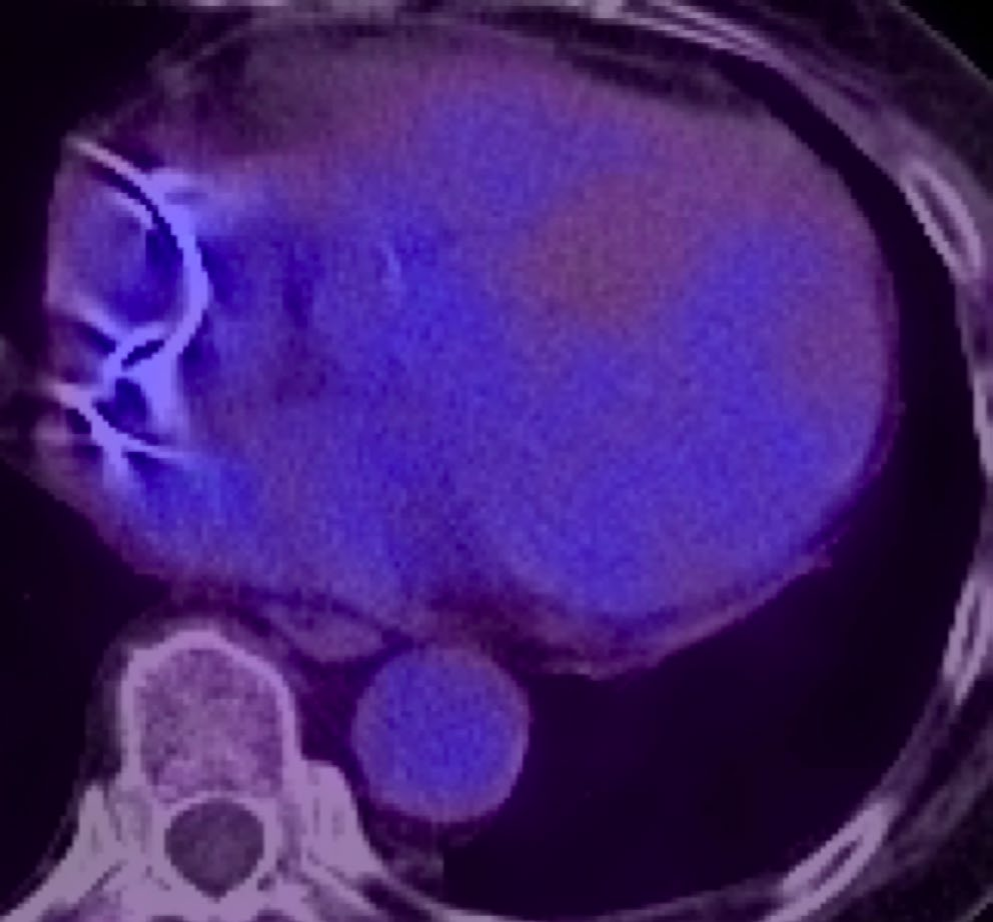
Repeated PET 11 months later (decreased cardiac uptake value)

We started prednisolone 30 mg daily with scheduled tapering and the multifocal uptake on PET significantly improved, as seen on the 11 month follow‐up images (Figure 7). Currently, the patient is being followed in the outpatient clinic and there has been no recurrent heart failure or ventricular arrhythmia. Her ECG shows no sign of AVB, and the intrinsic QRS wave is narrow. Right ventricular pacing burden was significantly improved from 95.7% just after PPM implantation to less than 1% at 12 months. The latest TTE demonstrated preserved biventricular systolic function.

## 2 DISCUSSION

### 2.1 Discrepancies among international guidelines: histopathological evidence in the early stages of CS

As described above, CS remains a life‐threatening condition compared to sarcoidosis involving other organs and diagnosis of early stage CS is challenging. There are three international guidelines for the management of CS: the 2017 Japanese Circulation Society (JCS) guideline,^1^ the 2014 Heart Rhythm Society (HRS) expert consensus statement,^2^ and the 2014 World Association of Sarcoidosis and Other Granulomatous Diseases (WASOG) Sarcoidosis Organ Assessment Instrument.^3^

Pulmonary biopsy is the most common site for extracardiac biopsy. Dziedzic et al reported the overall sensitivity of EBUS‐TBNA for stage I and II sarcoidosis was 84%, which is superior to transbronchial biopsy.^4^ However, as demonstrated in this case, early stage disease may lead to false negative results.^4^

If extracardiac tissue biopsies are negative, EMB is required to confirm myocardial involvement. The CS lesions tend to spread focally in the affected organ and obtaining appropriate specimens is technically difficult.^2^ The diagnostic accuracy of EMB is less than 20%‐25%.^1^, ^2^ As in our case, optimal timing of EMB is still uncertain in the setting of temporary pacing lead placement and EMB is frequently avoided in the peri‐procedural period.^2^, ^5^ Killu et al recently reported that EMB can be performed safely at the time of catheter ablation or cardiac implantable electronic device (CIED) implantation. However, their EMBs yielded diagnosis in only 28% of cases and there were technical limitations in obtaining appropriate EMB specimens.^5^

Even in the absence of EMB evidence, HRS and WASOG suggest clinical findings can indicate myocardial manifestation, if (a) extracardiac sarcoidosis has already been confirmed histologically and (b) other etiologies have been reasonably excluded.^2^, ^3^ However, as far as requiring pathological evidence, this approach could potentially overlook early stage CS.^1^

Of note, the revised JCS guideline enables clinicians to make CS diagnosis clinically without any histopathological evidence of sarcoidosis.^1^ To establish “clinically‐diagnosed CS,” patients must have both “CS‐specific cardiovascular findings” and histopathological/clinical diagnosis of extracardiac sarcoidosis such as pulmonary and/or ophthalmologic manifestations.^1^ CS‐specific cardiovascular findings are divided into primary and accessory findings. Primary findings include (I) high‐grade AVB and/or ventricular tachycardia/fibrillation, (II) CS‐specific TTE findings (basal interventricular septum wall thinness or ventricular aneurysm), (III) left ventricular systolic dysfunction or regional wall motion abnormality, (IV) abnormal extensive uptake in the myocardium with PET or Gallium scans, and (V) LGE with CMR. The accessary findings include (VI) non‐sustained ventricular tachycardia or multifocal premature ventricular contractions and (VII) focal deficit with single‐photon emission computed tomography (SPECT).^1^ At least two primary findings or one primary finding with two accessory findings are required for CS‐specific cardiovascular findings. In addition, clinicians must exclude other lymphoproliferative disease, malignant lymphoma, and tuberculosis.^1^ Besides clinical evidence of pulmonary manifestation such as BHL on CXR and PET, our patient fulfilled the criteria with three primary findings; high‐grade AVB, abnormal extensive uptake in the myocardium on ^18^F‐FDG‐PET, and LGE on CMR.

These sarcoidosis‐specific findings involving multiple organs are very important to differentiate CS from other etiologies such as lymphocytic, eosinophilic, or giant cell myocarditis. The most important differential diagnosis in CS is giant cell myocarditis (GCM). Although both CS and GCM have similar presentations such as acute heart failure or advanced arrhythmias, GCM is more rapidly progressive with higher mortality than CS. Several features of the disease course which Okura et al reported help us differentiate CS from GCM.^6^ For instance, the duration from symptomatic onset to hospital admission was more rapid for GCM than CS (1.2 ± 4.4 vs 5.5 ± 12.1 months, *P* < 0.01) and left‐sided heart failure was more common in GCM than in CS (64% vs 40%, *P* < 0.001), but AVB was more common in CS than in GCM (50% vs 15%, *P* < 0.001).^6^

Differentiating CS from GCM without histopathological evidence is still challenging but the new JCS guideline enables clinicians to diagnose early stage CS promptly and start urgent treatments. As there is no international consensus to date, it is too early to assess the diagnostic accuracy of this new strategy and a multicenter prospective study is required for further validation.

### 2.2 Appropriate utilization of imaging modalities for CS diagnosis

Our case highlighted the discrepancy between TTE and other imaging modalities in the early stage CS. The correct diagnosis of CS is still challenging in patients with seemingly normal left ventricular function on TTE. STE has been reported to be useful in detecting abnormal findings in these patients.^7^ Orii et al reported that circumferential strain could detect regional myocardial damage corresponding to LGE in CMR with high accuracy.^8^ However, even in cases with obvious LGE in CMR and/or uptake in PET as in our case, STE cannot necessarily detect abnormalities. Therefore, we want to highlight that unremarkable TTE/STE findings cannot exclude early stage CS. Clinicians must understand the limitations of echocardiography and continue aggressive evaluation with other imaging modalities.

Formerly, a high‐rate of false positive or inconclusive results in PET‐CT of CS was reported and an adequate suppression of physiological FDG uptake in myocardial muscle is very important for optimizing diagnostic accuracy. Currently, prolonged fasting of at least 12 hours with fatty rich and low carbohydrate meals the day before PET is indicated.^9^

### 2.3 Management of CS: benefit of appropriate immune‐therapy in early stage disease

Currently, all CS guidelines recommend both CIED and immune‐suppression therapy for management of CS‐induced AVB.^1^, ^2^, ^3^ Despite the potential reversibility of AVB, all CS guidelines strongly recommend permanent CIED implantation due to the unpredictable disease course.^1^, ^2^, ^3^

Therefore, the primary role of immune‐suppression is secondary prevention of cardiac dysfunction and ventricular arrhythmias. Efficacy of corticosteroid therapy is enhanced in early stage CS with well‐preserved ventricular systolic function.^10^, ^11^ However, the urgent initiation of immune‐suppression is often delayed due to the technical difficulty of obtaining histopathological evidence required in conventional guidelines. Hence, the revised JCS guideline will revolutionize the comprehensive management of CS.

## 3 CONCLUSION

Clinicians who manage sarcoidosis, primarily pulmonologists, and cardiologists, should familiarize themselves with these diagnostic paradigm shifts and consider early aggressive immune‐suppression and CIED utilization to minimize irreversible organ damage.

## CONFLICT OF INTEREST

All authors declare that they have no conflict of interest.

## AUTHOR CONTRIBUTION

All authors participated in drafting the article and revising it critically for intellectual content. All authors: interpreted data. TT, SS, MO, HS: provided medical care. HS and KH: pacemaker implantation and optimization. IC: provided extensive editing of this paper and advice on the literature summary.
